# NR4A1 expression aberrations contribute to radiotherapy resistance in gastric cancer

**DOI:** 10.1038/s41598-025-20348-4

**Published:** 2025-10-17

**Authors:** Peidong Ni, Shiyang Dong, Guozhong Yao, Zhengyuan Yan, Jiang Yan, Xu Zhang, Yu Shen, Yun Dai, Chuming Zhu

**Affiliations:** 1 Department of General Surgery, Xuzhou Central Hospital, Affiliated Hospital of Southeast University, Xuzhou, Jiangsu Province P.R. China; 2 Department of Thoracic Surgery, Fuyang Tumour Hospital, Fuyang, Anhui Province China; 3 Department of General Surgery, Liyang People’s Hospital, Liyang, Jiangsu Province China; 4 Department of General Surgery, Nanjing Lishui People’s Hospital, Nanjing, Jiangsu Province China; 5 Department of Pathology, Liyang People’s Hospital, Liyang, Jiangsu Province China

**Keywords:** Gastric cancer, Radioresistance, NR4A1, Gastrointestinal cancer, Tumour biomarkers

## Abstract

Gastric cancer (GC) is a common and life-threatening malignancy. It is often diagnosed at advanced stages. A major challenge in treatment is the development of radiotherapy resistance. This resistance significantly reduces the effectiveness of therapy. Therefore, our objective is to pinpoint genes linked to the resistance against radiotherapy in gastric cancer. The NR4A1 expression exhibited abnormalities in radioresistant gastric cancer cells, specifically MKN45-R and AGS-R cells, in comparison to their respective parental cells. Through high-throughput sequencing, we screened these cells and utilized quantitative reverse transcription-polymerase chain reaction analysis (qRT-PCR) to quantify NR4A1 expression. Functional analyses of NR4A1 in GC radioresistance were conducted by overexpressing NR4A1 in both MKN45-R and AGS-R cells, as well as their parental counterparts. Finally, the clinical relevance of NR4A1 in GC was confirmed through animal experiments. The expression of NR4A1 was significantly reduced in radioresistant cells, namely MKN45-R and AGS-R, as opposed to their parental counterparts. Overexpressing NR4A1 in MKN45-R and AGS-R cells resulted in heightened sensitivity to radiation. Additionally, NR4A1 overexpression exerted inhibitory effects on the growth and metastasis of GC tissues in animal models following irradiation (IR). Our observations indicate that NR4A1 plays a crucial role in enhancing the radiation sensitivity of gastric cancer cells, underscoring the therapeutic potential of NR4A1 in GC treatment.

**Supplementary Information:**

The online version contains supplementary material available at 10.1038/s41598-025-20348-4.

## Introduction

Gastric cancer (GC) stands as one of the predominant malignancies worldwide, imposing a substantial burden on mortality^1^. Surgery is a common approach for managing early GC; however, for patients with locally advanced and advanced tumors, relying solely on surgery may prove insufficient^2^. In clinical settings, locally advanced and advanced GC typically necessitate a multimodal treatment strategy, involving approaches such as surgery followed by chemotherapy, chemoradiotherapy, perioperative chemotherapy, or chemoradiotherapy^3^. Unfortunately, more than 50% of patients experience locoregional recurrence following curative resection. The treatment paradigms for GC have evolved over the years, with the gradual exploration of radiotherapy in its treatment. However, the outcomes of radiotherapy are not consistently satisfactory^4–8^. While efforts have been made to identify molecular determinants of radioresistance, the underlying mechanisms remain incompletely understood, highlighting a critical gap in knowledge that must be addressed to improve therapeutic outcomes. Until now, there has been limited research on the molecular mechanism of radioresistance in GC. Therefore, gaining a deeper understanding of the molecular mechanisms underlying radioresistance in GC holds significant clinical importance.

Nuclear receptors, a family of transcription factors, have become crucial molecular regulators implicated in diverse processes linked to carcinogenesis. These processes, such as apoptosis, DNA repair, proliferation, and migration, collectively contribute to the complexity of cancer development. Nuclear receptor subfamily 4 group A member 1 (NR4A1, also termed Nur77, TR3) is a critical transcription factor within this family. Numerous studies have demonstrated the involvement of NR4A1 in various tumor processes, where it often acts as a tumor suppressor. This involvement spans across various cancers, including gastric cancer, acute myeloid leukemia (AML), breast cancer, and metastatic ovarian cancer^9–11^. For instance, elevated NR4A1 expression in transgenic mice results in thymocyte apoptosis, and simultaneous knockout of NR4A1 and related nor-1 genes in transgenic mice leads to lethal myeloid leukemia^9^. Intriguingly, recent studies have implicated NR4A1 in therapy resistance across various cancer types. For instance, in prostate cancer, NR4A1 downregulation has been associated with docetaxel (DTX) chemoresistance^12^ while in hepatocellular carcinoma, its overexpression sensitized tumors to radiation^13^. These findings suggest that NR4A1 may function as a nodal point in therapeutic response pathways. However, its specific role in GC radioresistance has not been systematically investigated, leaving a crucial gap in both mechanistic understanding and potential translational applications.

This investigation induced radioresistance in the GC cell lines MKN45 and AGS, leading to the development of radioresistant variants, denoted as MKN45-R and AGS-R, respectively, through a gradual exposure to escalating doses of irradiation (IR). High-throughput sequencing techniques were employed to acquire mRNA expression profiles associated with radioresistance. Furthermore, quantitative reverse transcription-polymerase chain reaction (qRT-PCR) and Western blot analyses demonstrated a notable reduction in NR4A1 expression in MKN45-R and AGS-R in comparison to their parental cells. Hence, the objective of this article is to investigate the function and mechanism of NR4A1 in the context of radiotherapy resistance in GC.

## Materials and methods

### Cell culturing and generation of radioresistant MKN45-R and AGS-R cells

The MKN45 and AGS human GC cell lines employed in this research were sourced from the Cell Bank of the Chinese Academy of Sciences (Shanghai, China). Exponential growth-phase MKN45 and AGS cells were plated at a density of 1 × 10^5^ cells per dish and exposed to 2 Gy of ionizing radiation (IR) when their confluence reached approximately 70%. The IR was delivered using a medical linear accelerator (Precise accelerator, Elekta, UK). Following the initial radiation exposure, the surviving cells were cultured and passaged twice before undergoing an additional 2 Gy of radiation. The cells were subjected to a cumulative dose of 60 Gy of ionizing radiation (IR) through a gradient irradiation protocol, with doses of 2, 4, 6, and 8 Gy administered in two cycles. The entire selection process took approximately 5 months. The ultimately surviving MKN45 and AGS cells were validated and designated as radioresistant MKN45 and AGS cells, denoted as MKN45-R and AGS-R. Experiments were carried out utilizing MKN45-R and AGS-R cells in passages 4–10 post the final round of ionizing radiation (IR) exposure. Control MKN45 and AGS cells underwent the same treatment regimen excluding irradiation. MKN45, AGS, MKN45-R, and AGS-R cells were cultured in RPMI-1640 medium supplemented with 10% fetal bovine serum (Wisent, Montreal, Canada) and 1% antibiotics (100 U/ml penicillin G and 100 mg/ml streptomycin), and maintained at 37 °C in a humidified 5% CO₂ incubator. All experiments were performed using exponentially growing cells.

### The experiment of cell viability assay

Cell proliferation was assessed using the Cell Counting Kit-8 (CCK-8) (Vazyme, Nanjing, China) according to the manufacturer’s recommendations. A seeding density of 2000 cells per well was employed in 96-well plates, and the cells were cultured for a duration of 5 days. At designated time intervals each day, CCK-8 reagent was added to the respective plates, and the cells were incubated for 2 hours at 37 °C. The absorbance at 450 nm was subsequently measured to evaluate the cell proliferation capacity. Data from the CCK-8 assay were analyzed using independent two-sample t-tests, with statistical significance set at *p* < 0.05. GraphPad Prism (Version 8.0, GraphPad Software, San Diego, CA, USA) was used for data analysis. The parameters α, β, SF2, and MID were computed using the linear-quadratic (LQ) model, whereas D0, Dq, N and R^2^ were obtained through a multi-target, single-hit model.

### Colony formation assay

The colony formation assay was employed to assess the impact of different cell types on proliferation in response to IR. As previously described, cells were seeded in 6-well culture plates at varying densities. Approximately 24 h after cell attachment, the cells underwent irradiation with doses ranging from 0 to 8 Gy. Following that, all cells were subjected to staining with 1% crystal violet (Beyotime, Shanghai, China) after a 14-day incubation period. Colonies, each comprising 50 or more cells, were tallied and photographed. Plating efficiencies (PE) were determined by dividing the number of colonies by the initial number of seeded cells. The survival fraction was calculated by dividing the PE of the irradiated group by that of the non-irradiated group. All experimental procedures were performed in triplicate.

### Flow cytometric assessment of cellular apoptosis in reaction to radiation

Flow cytometric analysis was utilized to evaluate the proportion of cells undergoing apoptosis. The detailed experimental procedures are outlined in the preceding Sect^14^.. All experiments were performed in triplicate.

### Quantitative real-time polymerase chain reaction (qRT-PCR)

Standard TRIzol RNA extraction protocol (Invitrogen, Carlsbad, CA, USA) was employed to extract total RNA. Following total RNA extraction using the standard TRIzol protocol (Invitrogen, Carlsbad, CA, USA), cDNA synthesis was performed using PrimeScript RT Reagent (TaKaRa, Japan). Subsequent quantitative real-time polymerase chain reaction (qRT-PCR) was conducted on a 7500 Real-time PCR System (Applied Biosystems, Carlsbad, CA, USA) with AceQ qPCR SYBR^®^ Green Master Mix (Vazyme, Biotech, Co.,Ltd). The expression level of GAPDH was employed as the internal control for NR4A1. The primer sequences utilized in this study were as follows: NR4A1 forward sequence, 5′-CACCCACTTCTCCACACCT-3′, and reverse forward sequence, 5′-TCCCCACACACAGCACA-3′. For GAPDH, the forward primer sequence was 5′-TGCACCACCAACTGCTTAGC-3′, and the reverse primer sequence was 5′-GGCATGGACTGTGGTCATGAG-3′. The manufacturer’s instructions were followed at each step.

### Western blotting analysis

Western blot assays were performed under standard conditions^15^. The primary antibodies utilized are detailed in Supplementary Table 1.

### Cell transfection

The NR4A1 overexpression plasmid and corresponding negative controls were designed and synthesized by GenePharma (Shanghai, China). Transfection was performed using Lipofectamine 3000 Reagent (Invitrogen, USA) according to the manufacturer’s recommendations. After 48 h of transfection, the efficiency of transient transfection was evaluated through qRT-PCR analysis.

### Cell migration and invasion assays

A 6.5 mm chamber with 8 μm pores (Corning Costar Corp., USA) was used to assess the migratory and invasive abilities of GC cells. For the migration assay, 2 × 10^4 transfected GC cells were seeded into the upper chamber containing 200 µl of serum-free RMPI-1640 medium. The lower chamber was supplemented with 500 µl of RMPI-1640 medium enriched with 10% fetal bovine serum (FBS), serving as a chemoattractant. Following a 24-hour incubation period, cells that had traversed to the lower surface were fixed and stained with 1% crystal violet for 30 min.

In parallel, invasion assays were conducted by pre-coating the upper chamber with 0.1 ml of Matrigel (at a concentration of 50 µg/ml, BD Biosciences, USA) prior to cell seeding. The subsequent procedural steps were identical to those of the migration assay. All experiments were executed in triplicate to ensure reproducibility of results.

### Animal experiments

In the tumor xenograft model, 20 female nude mice were randomly assigned to four groups: NR4A1-vector-NC, oe-NR4A1-NC, NR4A1-vector-Radiotherapy, and oe-NR4A1-NC. Stable cells (1 × 10^6^ cells/100 µl of PBS) were injected into the flanks of the nude mice in their respective groups. Tumor volume was assessed every 4 days and calculated using the formula: volume = (length × width ^ 2)/2. Upon reaching a tumor volume of 50 mm^3, four fractions of radiation (2 Gy/f) were administered to the tumors, and the mice were sacrificed 14 days after the completion of radiation exposure. Lead shields were employed to mitigate radiation injury.

For the metastasis model, the remaining 12 mice were randomly assigned to four groups: vector-NC, oe-NR4A1-NC, vector-Radiotherapy, oe-NR4A1-Radiotherapy, (*n* = 3/group). Stable cells (1 × 10^6^ cells/100 µl of PBS) were intravenously injected into the tail vein of mice.

After 5 weeks, the occurrence of distant metastasis was monitored using an IVIS imaging system (Caliper Life Sciences, Hopkinton, MA, USA). Isoflurane was used for euthanasia. Mice were exposed to a high concentration of isoflurane (5% − 6% for induction in a closed chamber, followed by maintenance at 3% − 4% until death). This concentration was maintained until the absence of heartbeat and respiratory movements, as well as fixed and dilated pupils, confirmed the death of the animals.

This study received approval from the Institutional Animal Care and Use Committee of Southeast University (Approval No.20220208009). All experiments described in this manuscript were conducted in strict accordance with relevant guidelines and regulations. In addition, this study is reported in full compliance with the ARRIVE (Animal Research: Reporting of In Vivo Experiments) guidelines.

### Immunohistochemistry

All specimens, comprising GC tissues and their paired normal counterparts, were initially preserved in 4% formaldehyde before being encased in paraffin. Subsequent to the inhibition of endogenous peroxides and proteins, sections measuring 4 micrometers were subjected to an overnight incubation at 4 °C with primary antibodies targeting Ki-67, sourced from Abcam. Following a thorough PBS rinse, the slices underwent a 1-hour incubation at 37 °C with an HRP-conjugated secondary antibody and were then treated with a 3,3’-diaminobenzidine solution for 3 min to render the staining visible. Nuclei received a counterstain of hematoxylin. The tumor sections were examined blindly. Three disparate fields were chosen at random to gauge the proportion of tumor positivity and the intensity of cellular staining.

### Statistical analysis

Every experiment was conducted independently a minimum of three times. The experimental data are presented as mean ± standard deviation (SD) and were statistically analyzed using GraphPad Prism 8.0. Pearson χ2 tests were employed for the comparison of clinicopathological results. The comparison between the treated group and the control group was conducted using Student’s t-test and ANOVA. Statistical significance was defined as a p-value < 0.05.

## Results

### MKN45-R and AGS-R radiation-resistant cells are established and the radiation-resistant ability of MKN45-R is verified

To establish a representative preclinical in vitro cell model, this study employed the classic gastric cancer cell lines MKN45 and AGS. The parental MKN45 and AGS cells underwent exposure to a segmented dose gradient of 2, 2, 4, 4, 6, 6, 8, 8, 10, 10 Gy of radiation, and the whole selection procedure was completed over 5 months. For the generation of a stable and viable resistant cell line, cells underwent meticulous culturing and stable passaging. When subjected to the dual influence of tumor circulation and radiation, cells underwent a dynamic process involving apoptosis, survival, and the development of resistance with an increase in accumulated radiation dose. Ultimately, radiation-resistant cells became the predominant component.

**Fig. 1:**
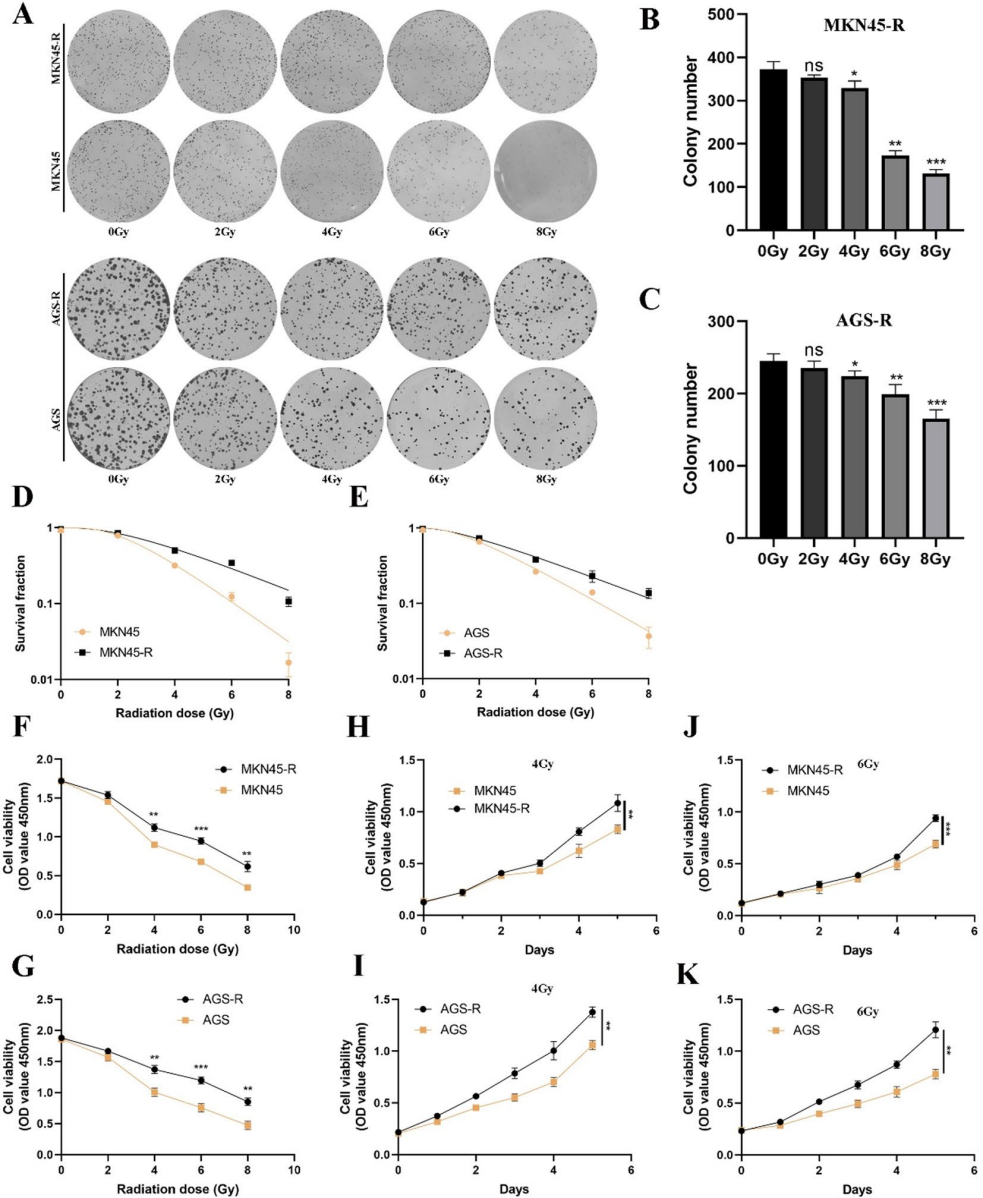
Radioresistant MKN45 and AGS cells with radioresistance (MKN45-R AGS-R) cells are established and validated. (**A**, **B** and **C**) Colony formation assay to evaluate the proliferation capacity of MKN45-R and AGS-R cells and their parental cells under different radiation doses. (**D** and **E**) Survival curve data of MKN45-R and AGS-R with their parental cells. (**F**-**K**) CCK-8 assay to evaluate the proliferation capacity of MKN45-R and AGS-R cells and their parental cells after exposure to different radiation doses. Statistical analysis was performed by SPSS software using Student’s t-test. *p < 0.05, **p < 0.01, and ***p < 0.001 indicated significant differences.

To assess the impact of radiation dose on the radiation sensitivity of cells, we initially conducted a cell clone formation experiment. The results revealed that post-radiation, MKN45-R and AGS-R cell colonies exhibited increased survival, with larger colonies compared to their parental counterparts (Fig. 1A-C, Supplementary Fig. 1A-B). Furthermore, we investigated the effect of ionizing radiation (IR) on the growth of GC cells by subjecting MKN45-R and AGS-R, along with their parental cells, to varying doses of radiation. Illustrated in Fig. 1F-K, MKN45-R and AGS-R cells, along with their parental counterparts, exhibited comparable growth patterns in the absence of ionizing IR exposure. Nevertheless, upon exposure to IR at 4 Gy or higher, a significant divergence in cell growth became evident. The survival curve data were further analyzed using an improved linear multi-target single-hit model, yielding radiobiologically relevant parameters (Fig D-E). As shown in Table 1, in radioresistant (RR) cells, the radioresistant indicators - including the 37% dose slope (D0), quasi-threshold dose (Dq), mean inactivation dose (MID), and surviving fraction at 2 Gy irradiation (SF2) - were all increased.

**Table 1:** Comparison of survival curve model parameters between parental cells (MKN45/AGS) and radioresistant cell lines (MKN45-R/AGS-R).

Cell lines	D_0_	Dq	*N*	α/β	SF2	MID	*R* ^2^
MKN45	1.619	2.421	4.460	0.899	0.502	2.48	0.982
MKN45-R	2.812	2.793	2.700	5.314	0.599	3.31	0.977
AGS	2.021	1.645	2.257	4.108	0.636	3.54	0.990
AGS-R	2.992	1.654	1.738	9.438	0.693	4.49	0.991

### NR4A1 expression is decreased in MKN45-R and AGS-R cells compared to the parental cells

**Fig. 2:**
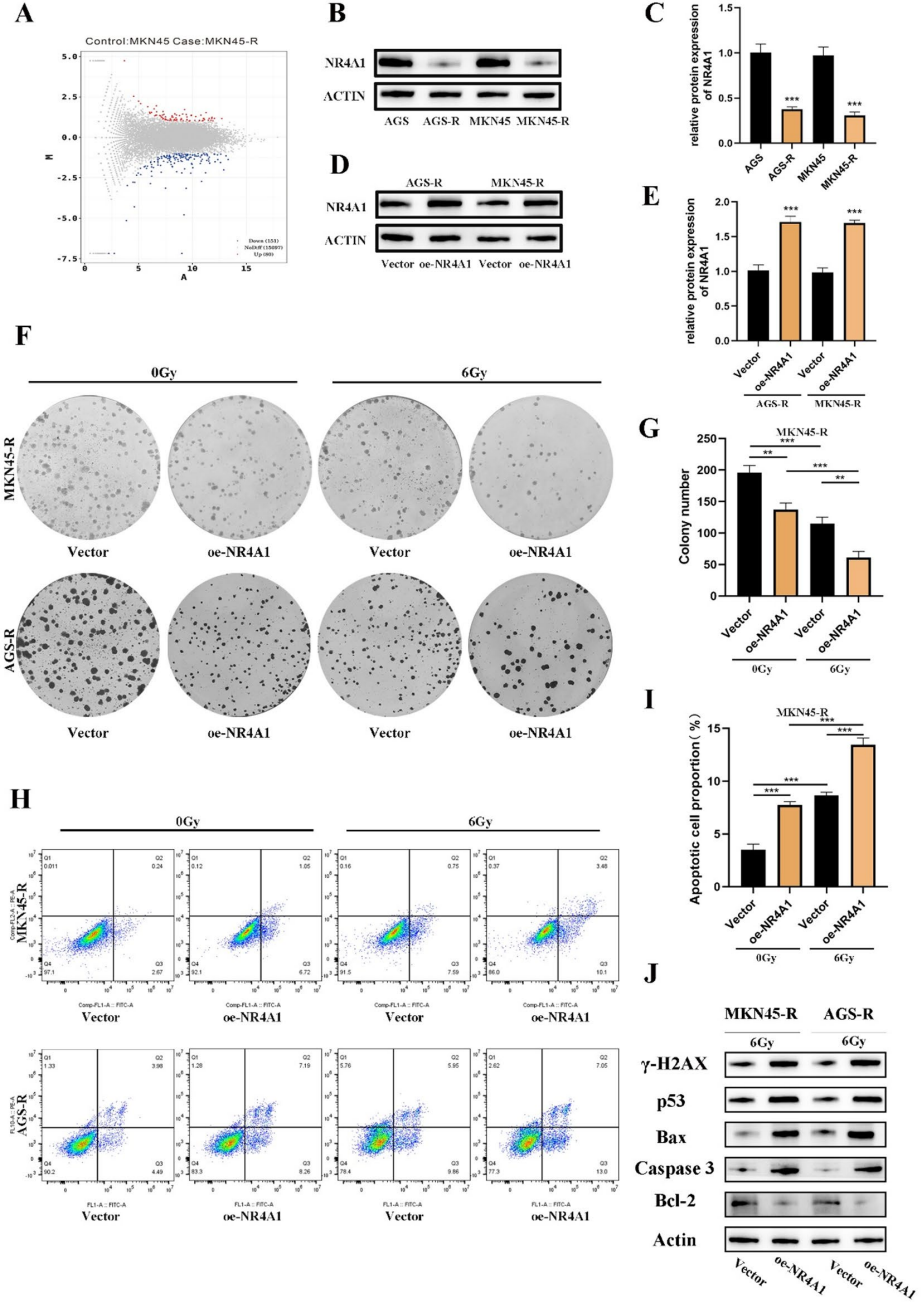
NR4A1 overexpression in MKN45-R and AGS-R cells results in increased sensitivity to irradiation. (**A**) The expression of NR4A1 in the radioresistant MKN45-R and AGS-R cells and their parental cells was analyzed by second-generation sequencing technology. (**B**-**E**) The expression of NR4A1 in the radioresistant cells and their parental cells was detected by quantitative reverse transcription-polymerase chain reaction (qRT-PCR) and Western blot analysis. (**F** and **G**) A representative image of colony formation assay by crystal violet staining in MKN45-R and AGS-R gastric cancer cells and their parental cells with or without 6 Gy X-ray irradiation. (**H**-**I**) Apoptosis of GC cells MKN45 AGS and radioresistant cells MKN45-R AGS-R after exposure or non-exposure to 6 Gy X-ray irradiation for 72 h. Apoptosis was detected by flow cytometry using Annexin V-FITC/PI staining. (**J**) Changes in the expression levels of the γ-H2AX, P53, Bax, Caspase and Bcl2 was detected by Western blot. The results were expressed as the mean ± S.D of three independent experiments. Statistical analysis was performed by SPSS software using Student’s t-test. *p < 0.05, **p < 0.01, and ***p < 0.001 indicated significant differences.

We employed high-throughput mRNA sequencing to delineate differential mRNA expression profiles between radioresistant cells and their parental counterparts. Our results revealed 151 downregulated mRNAs and 80 upregulated mRNAs, taking into account both a fold change > 1.0 and *p* < 0.05 (Fig. 2A). Among these expression profiles, specific differentially expressed genes associated with tumor occurrence or development, including the acquisition of radiation resistance, were identified. This observation suggests the potential contribution of differentially expressed genes to the acquired radioresistance in GC, warranting further investigation. Despite the limited research on NR4A1 in tumor biology, our study indicates a significant downregulation of NR4A1 in radiation-resistant cell lines compared to their parental cells, demonstrating a significant reduction of approximately 2.5-fold, validated by qRT-PCR and Western blot experiments (Fig. 2B-C, Supplementary Fig. 1 C). This finding hints at a potential role for NR4A1 in acquired radiation resistance in GC, necessitating further in-depth exploration.

### The ectopic expression of NR4A1 in MKN45-R and AGS-R cells can enhance their irradiation sensitivity

By inducing overexpression of NR4A1 in radioresistant GC cells, specifically MKN45-R and AGS-R, our aim is to explore the potential role of NR4A1 in the radiation biology of GC. In an in vitro experimental approach, we introduced an overexpression vector containing NR4A1 into the RR cells MKN45-R and AGS-R, alongside a corresponding control vector. The successful elevation of NR4A1 expression was confirmed through quantitative reverse transcription-polymerase chain reaction (qRT-PCR) and Western blot analyses (Fig. 2D-E, Supplementary Fig. 1D). Subsequently, in a colony formation assay, it was observed that the survival rate of MKN45-R and AGS-R cells with NR4A1 overexpression was markedly diminished compared to the control cells upon exposure to 6 Gy radiation (Fig. 2F-G, Supplementary Fig. 1E). The assessment of cellular apoptosis demonstrated a significant increase in the apoptosis rate in MKN45-R and AGS-R cells overexpressing NR4A1 after exposure to 6 Gy irradiation, in comparison to the control cells (Fig. 2H-I, Supplementary Fig. 1 F). Western blot analysis revealed that NR4A1 overexpression markedly increased the protein expression levels of p53 and cleaved caspase-3, accompanied by a significant elevation in the Bax/Bcl-2 ratio (Fig. 2J). These findings collectively demonstrate that NR4A1 triggers apoptosis in irradiated cells, suggesting its critical role in enhancing radiation-induced cell death through activation of the intrinsic apoptotic pathway.

### NR4A1 facilitates GC cell metastasis in vitro and in vivo

**Fig. 3:**
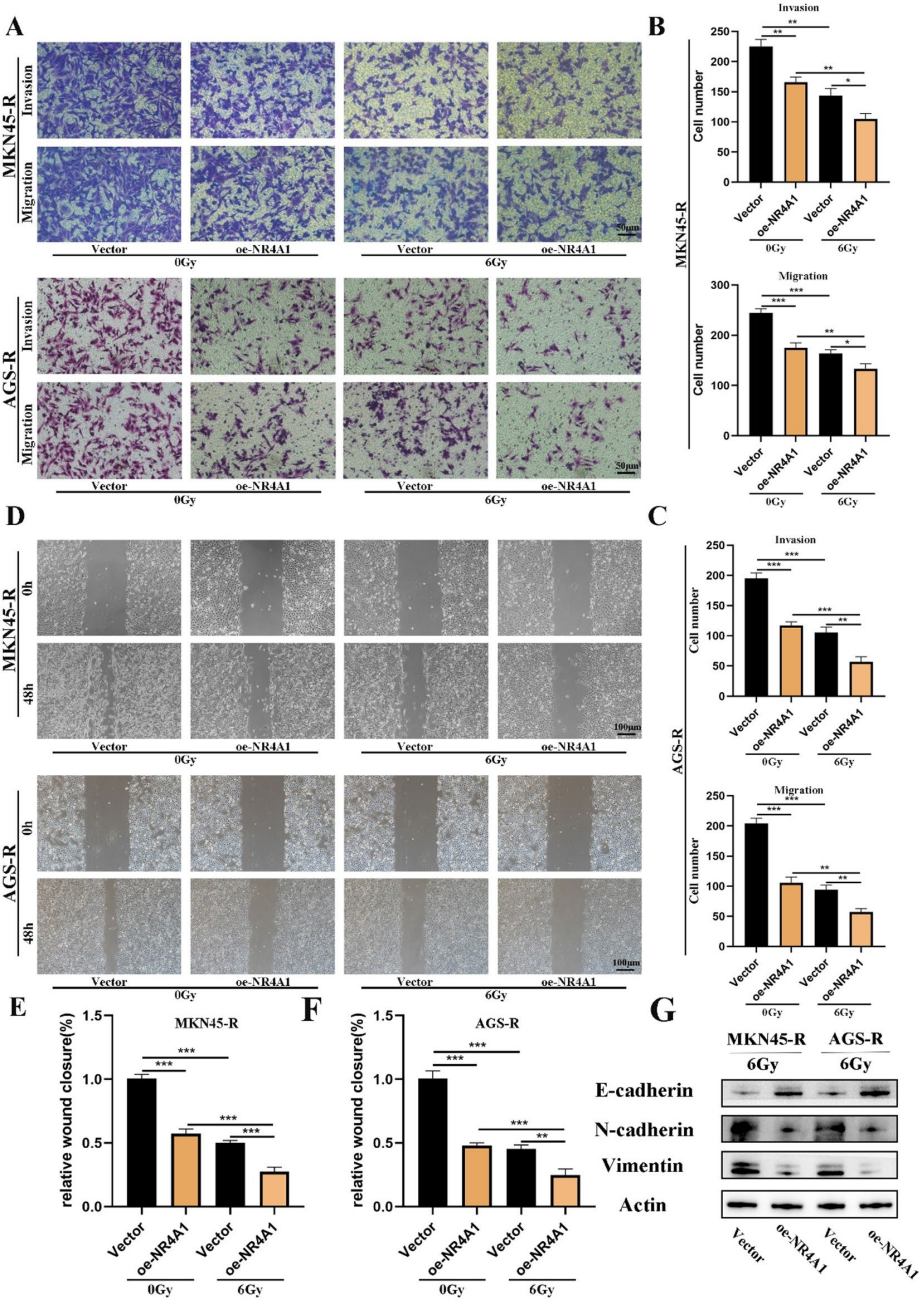
NR4A1 facilities migration and invasion of GC in vitro. (**A**-**C**) Transwell assays were performed under conditions with or without 6 Gy X-ray irradiation to examine the effects of NR4A1 on GC cell migration and invasion. (**D**, **E, F** and **G**) Wound healing assay for NR4A1 in GC cell migration without 6 Gy X-ray irradiation. Statistical analysis was performed by SPSS software using Student’s t-test. *p < 0.05, **p < 0.01, and ***p < 0.001 indicated significant differences.

Metastasis is a prominent hallmark of GC. During the cell culture process, we observed a distinctive behavior in RR cells, where they exhibited a tendency to disperse from each other during growth, in contrast to their parental cells. In our study, we explored the correlation between NR4A1 gene expression and the in vitro metastatic capacity of GC cells. Transwell experiments unveiled a significant reduction in the invasive capacity of cells overexpressing NR4A1 in both AGS-R and MKN45-R (Fig. 3A-C). Additionally, in wound healing experiments, the migration rate of the NR4A1 overexpression group showed a noticeable enhancement following exposure to 6 Gy radiation (Fig. 3D-F). These results present compelling evidence, prompting additional investigation into the involvement of NR4A1 in the migration of GC cells. Western blot analysis revealed that NR4A1 overexpression significantly upregulated E-cadherin expression while concurrently downregulating N-cadherin and vimentin protein levels in gastric cancer cells (Fig. 3G). These data collectively suggest that NR4A1 exerts a potent inhibitory effect on the epithelial-mesenchymal transition (EMT) process, as evidenced by the restoration of epithelial markers and suppression of mesenchymal markers.

### NR4A1 attenuated GC cell proliferation and metastasis in vivo

**Fig. 4:**
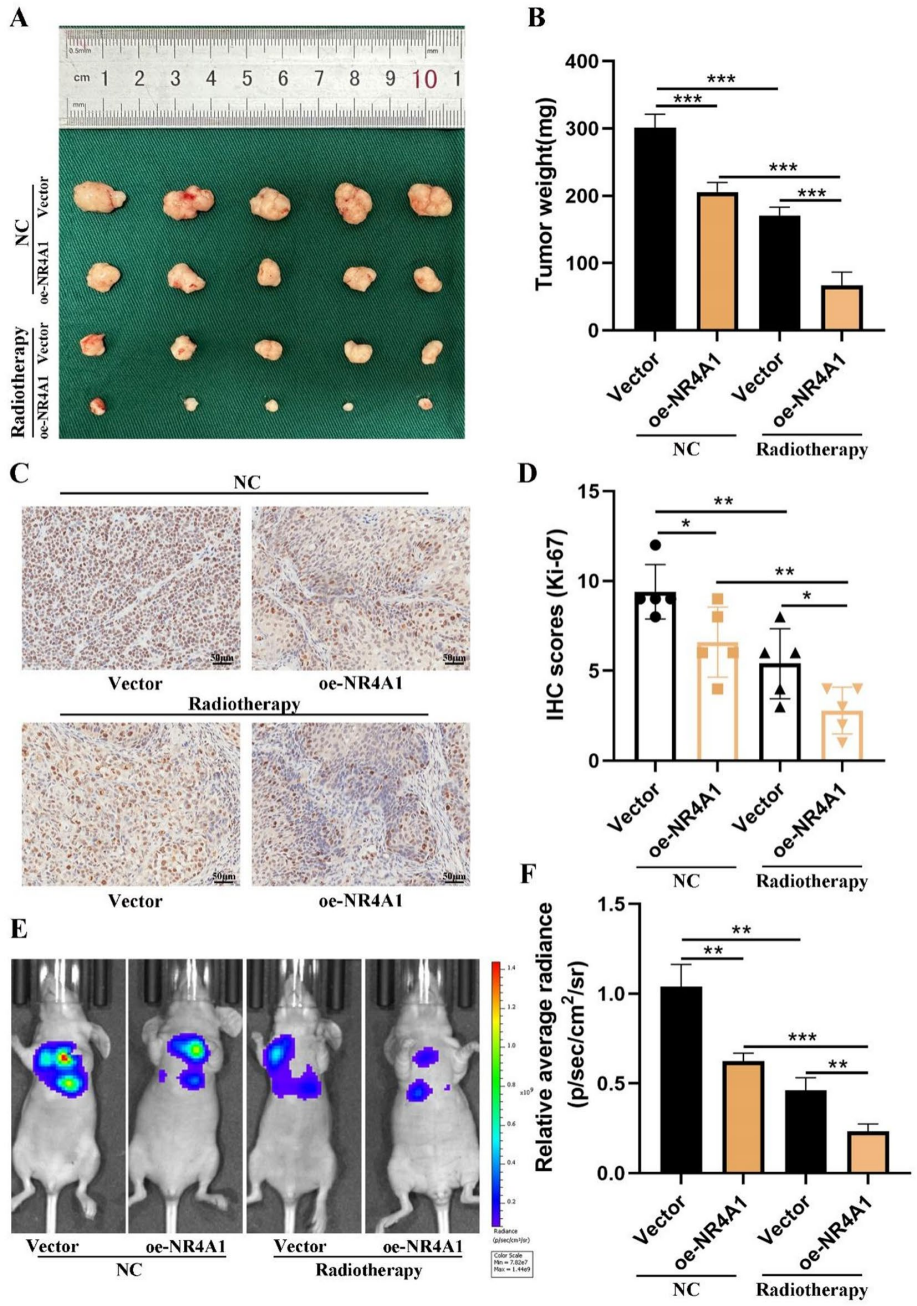
NR4A1 facilities the proliferation and metastasis of gastric cancer cells in vivo. (**A**-**D)** Tumor size after the tumors were harvested. Representative images of the expression and distribution of Ki-67 in gastric cancer tissue and corresponding normal tissue by IHC. Scale bar = 50 μm (left), scale bar = 25 μm (right). Error bars, mean ± SD. *P < 0.05; **P < 0.01. (**E** and **F**) Representative photographs of tumours were taken by IVIS Imaging System from different groups. Statistical analysis was performed by SPSS software using Student’s t-test. *p < 0.05, **p < 0.01, and ***p < 0.001 indicated significant differences.

To elucidate the in vivo effects of NR4A1, we established subcutaneous tumor and lung metastasis models. Evidently, within the subcutaneous model, the tumor weight exhibited a significant reduction in the NR4A1 overexpression group as opposed to the control group under radiation exposure (Fig. 4A-B). The NR4A1-overexpressing tumors exhibited lower levels of Ki-67 (Fig. 4C-D). Certainly, these results affirm the crucial role of NR4A1 in modulating the proliferation of GC cells under radiation exposure. Subsequently, we delved into the impact of NR4A1 on tumor metastasis. Stable transfected cells were intravenously injected into nude mice, and fluorescence signals were assessed after 5 weeks. The outcomes demonstrated a notable decrease in lung metastasis among mice exhibiting NR4A1 overexpression in comparison to the control group (Fig. 4E-F).

## Discussion

Gastric cancer (GC), a highly malignant disease, is frequently diagnosed at advanced stages, significantly limiting treatment options^16–18^. Recent diagnostic trends indicate that over three-quarters of patients with gastric cancer are identified at an advanced stage, characterized by tumor invasion into the muscularis propria or regional lymph nodes, correlating with survival probabilities ranging from 20% to 50%^19^. Surgery is no longer feasible for approximately half of these patients. Consequently, treatment strategies have increasingly focused on multimodal approaches encompassing radiotherapy (RT) and chemotherapy. The evolution of RT in the therapeutic landscape of GC has been notable, particularly following the seminal publication by John et al. in 2001 within the New England Journal of Medicine. The INT0116 trial delineated therein marked a paradigm shift, repositioning RT from a mere palliative approach to a critical adjunctive therapy within the framework of multidisciplinary management of GC^20^. This trial marked a paradigm shift, transitioning RT from a primarily palliative approach to a crucial adjunctive therapy within multidisciplinary GC management.

The clinical process of GC radiotherapy resistance is a critical aspect that warrants comprehensive exploration. Radioresistance can lead to local treatment failure and recurrence, driven by multiple factors. These include: radiation-induced DNA damage repair, cell cycle arrest, evasion of apoptosis, and the presence of cancer stem cells. Additionally, alterations in cancer cells and their microenvironment, exosomal and non-coding RNA activity, metabolic reprogramming, and ferroptosis contribute significantly to radioresistance^21,22^. Hence, there is a pressing need to investigate novel strategies to overcome GC resistance. In this context, it is significant that upregulation of NR4A1 has been shown to enhance the sensitivity of GC cells to RT.

In this investigation, we initially established two radioresistant cell lines. Subsequent in vitro and in vivo experiments revealed that NR4A1 was downregulated in RR cells. Simultaneously, the upregulation of NR4A1 was found to enhance the radiosensitivity of GC cells. The involvement of NR4A1 in cancer progression and metastasis has been thoroughly examined. NR4A1 knockout mice demonstrate a swift onset of acute myeloid leukemia, and NR4A1 is proficient in inducing apoptosis in GC cells^9,23^. Additionally, NR4A1 serves as a tumor suppressor, governing the malignant characteristics of cervical cancer cells and instigating apoptosis in cervical cancer cells^24^. In the context of therapeutic resistance, NR4A1 is transcriptionally repressed by MTA1 through its interaction with histone deacetylase 2 (HDAC2), thereby reducing chemosensitivity^12^. Notably, in hepatocellular carcinoma cells, radiation-induced suppression of NR4A1 was observed to diminish cellular radiosensitivity^13^. Interestingly, NR4A1-knockout murine models exhibited more severe radiation-induced cutaneous damage post-irradiation^25^. This phenomenon may stem from tissue-specific regulatory mechanisms, consistent with the paradoxical roles of NR4A1 across different biological systems. Correspondingly, this study delineates the role of NR4A1 in modulating radiation responses in GC cells, demonstrating that NR4A1 enhances radiosensitivity by augmenting radiation-induced apoptosis.

DNA double-strand breaks (DSBs) are widely recognized as the most lethal form of DNA damage and represent the primary cause of cell death induced by IR^26^. IR-induced DSBs can result in three distinct biological consequences: (1) Faithful repair, enabling cellular survival with restored genomic integrity; (2) Apoptotic elimination, occurring when DSBs exceed repair capacity, thereby preventing propagation of damaged DNA; (3) Error-prone repair or persistent damage, which may induce genomic instability and serve as a potential precursor to malignant transformation. In Wu’s study, NR4A1 can bind to p53 and block MDM2-mediated ubiquitination and degradation, thereby enhancing p53 stability^27^. NR4A1 physically interacts with Ku80 and inhibits its DNA end-binding activity, thereby delaying DSB repair in hepatocellular carcinoma cells. Meanwhile, NR4A1 directly suppresses the DNA end-binding activity of Ku80, preventing DNA-dependent protein kinase (DNA-PK) from binding to DNA^13^. However, DNA-PK can still phosphorylate NR4A1. This phosphorylation, in turn, enhances the effect of NR4A1 on IR-induced p53 transcriptional activity in a DNA-PK-dependent manner. A feedback loop may exist between DNA-PK and NR4A1 to modulate p53 phosphorylation. In this mechanism, DNA-PKcs phosphorylates NR4A1, and the modified NR4A1 then promotes DNA-PK-mediated p53 phosphorylation via protein interactions. In this study, we found that NR4A1 enhances p53 expression and significantly induces apoptosis in GC cells both with and without IR following IR. Therefore, we speculate that the DNA-PK and NR4A1 feedback loop may serve as a potential therapeutic target to overcome radioresistance in GC. Our preliminary data suggesting potential interactions between NR4A1 and radioresistance-associated genes present a novel avenue for understanding the mechanisms underlying radioresistance. However, how NR4A1 mediates its contradictory role in repression of repair factors with its own phosphorylation by DNA-PK remains to be elucidated.

To address this, the genomic binding profile of NR4A1 will be mapped by ChIP-seq to determine whether NR4A1 directly regulates p53 target genes such as BAX and CDKN1A. Site-specific mutagenesis assay was used to verify the effect of DNA-PK-mediated phosphorylation on NR4A1 function. In addition, the strategy of combining targeting DNA-PK with NR4A1, such as inhibitor + agonist, to test its efficacy in overcoming radioresistance in an in vivo model has significant translational value.

Metastasis is a critical life-threatening event in gastric cancer (GC) patients. The persistence of cancer cells driving metastatic progression remains a major therapeutic challeng^16,17^. GCs have been reported to display therapeutic resistance as well as a high metastatic capacity^15^. To investigate NR4A1’s potential role in metastasis, we intravenously injected GC cells overexpressing NR4A1 into mice. Notably, these mice exhibited significantly fewer pulmonary metastatic nodules compared to controls injected with non-overexpressing cells. These results suggest NR4A1 may suppress GC metastasis. Thus, further exploration of NR4A1’s role in GC metastasis holds promise for advancing cancer therapeutics.

This study has several limitations. We currently lack a comprehensive mechanistic understanding of how NR4A1 enhances the radiosensitivity of GC cells. Further research has been incorporated into our plan. Defining the genome-wide binding landscape of NR4A1 in gastric cancer cells and elucidating its paradoxical role in DNA repair mechanisms represent critical priorities for our future work. Moreover, due to the limited number of pathogens, we were unable to collect a sufficient number of tissue samples, resulting in a lack of corresponding clinical correlation analysis. We will continue to collect samples in the future, and the clinical relevance analysis will further confirm the practical significance of this research. Furthermore, clinical trials investigating NR4A1 as a radiosensitization target in GC radiotherapy also hold significant clinical importance. The combination of these approaches may potentially enable GC patients to achieve improved prognosis.

To summarize, our study outcomes indicate a crucial involvement of NR4A1 in the RR of GC cells. Therefore, focusing on this pathway specifically may provide a biologically driven strategy to enhance the effectiveness of radiotherapy. Following functional radiobiological assays and an analysis of NR4A1 in a retrospective cohort of patients treated with RT, there is potential for the identification of novel predictive biomarkers for radiation response in GC.

## Conclusion

In conclusion, the study indicates that the downregulation of NR4A1 induced by radiation might be a key factor in the development of radiotherapy resistance in gastric cancer. This discovery opens avenues for potential biomarkers and strategies to improve the prognosis of GC patients.

## Supplementary Information

Below is the link to the electronic supplementary material.

Supplementary Material 1

Supplementary Material 2

Supplementary Material 3

## Data Availability

All data generated or analyzed during this study are included in this article and available from the corresponding author upon reasonable request. Sequencing data supporting the findings have been deposited in the Gene Expression Omnibus (GEO) repository under accession number GSE286081.
