# Supplementary material for: NR4A1 expression aberrations contribute to radiotherapy resistance in gastric cancer
Source: Sci Rep. 2025 Oct 17;15:36394. doi: 10.1038/s41598-025-20348-4 (PMC12534420; doi:10.1038/s41598-025-20348-4)
Supplement: Supplementary file 1 — Supplementary Material 1 [file 41598_2025_20348_MOESM1_ESM.docx]

**Supplementary Table 1. Antibodies used for Western blot**

| antibody | company/provider(Dilution ratio) |
| --- | --- |
| β-actin | Proteintech (1:5000) |
| anti-NR4A1 | Proteintech (1:1000) |
| anti-Ki67 | Proteintech (1:16000) |
| γ-H2AX | Abcam (1:5000) |
| p53 | Proteintech (1:2000) |
| Bax | Proteintech (1:5000) |
| Caspase3 | Proteintech (1:1000) |
| Bcl-2 | Proteintech (1:1000) |
| E-cadherin | Proteintech (1:5000) |
| N-cadherin | Proteintech (1:2000) |
| Vimentin | Proteintech (1:3000) |
