# Supplementary material for: NR4A1 expression aberrations contribute to radiotherapy resistance in gastric cancer
Source: Sci Rep. 2025 Oct 17;15:36394. doi: 10.1038/s41598-025-20348-4 (PMC12534420; doi:10.1038/s41598-025-20348-4)
Supplement: Supplementary file 2 — Supplementary Material 2 [file 41598_2025_20348_MOESM2_ESM.docx]

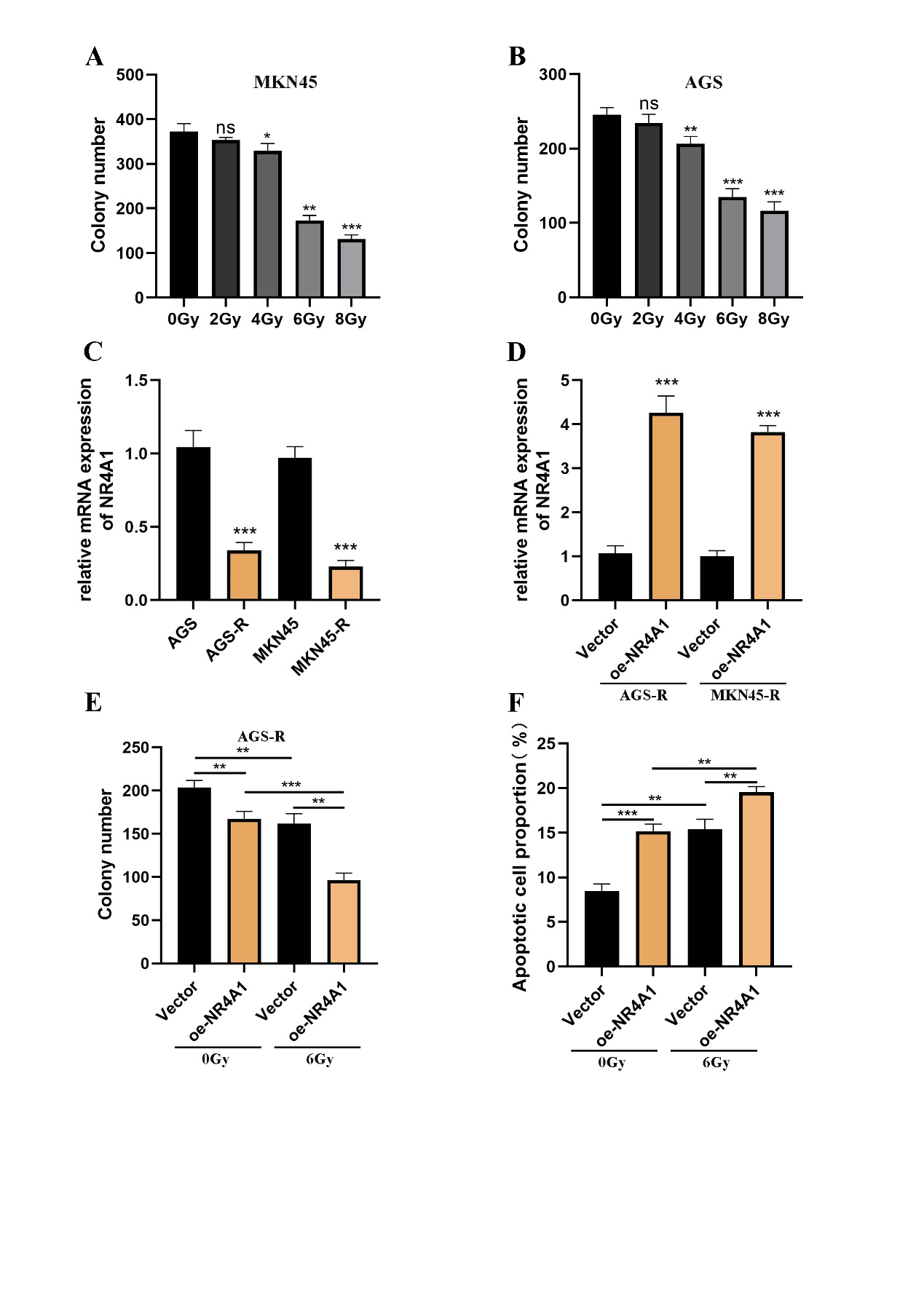
Supplementary Figure 1 A and B The number of colony formation of MKN45 and AGS cells decreased gradually with the increase of radiation dose.C The expression of NR4A1 was significantly reduced in the radioresistant cell lines MKN45-R and AGS-R compared with their parental cells. D The expression of NR4A1 was significantly increased in MKN45-R and AGS-R cells after overexpressing NR4A1. E The number of colony formation of AGS-R cells overexpressing NR4A1 was significantly reduced under 0Gy and 6Gy X-ray irradiation. F The proportion of apoptosis in AGS-R cells overexpressing NR4A1 was significantly increased under 0Gy and 6Gy X-ray irradiation.
