# Supplementary material for: NR4A1 expression aberrations contribute to radiotherapy resistance in gastric cancer
Source: Sci Rep. 2025 Oct 17;15:36394. doi: 10.1038/s41598-025-20348-4 (PMC12534420; doi:10.1038/s41598-025-20348-4)

Fig2B

NR4A1


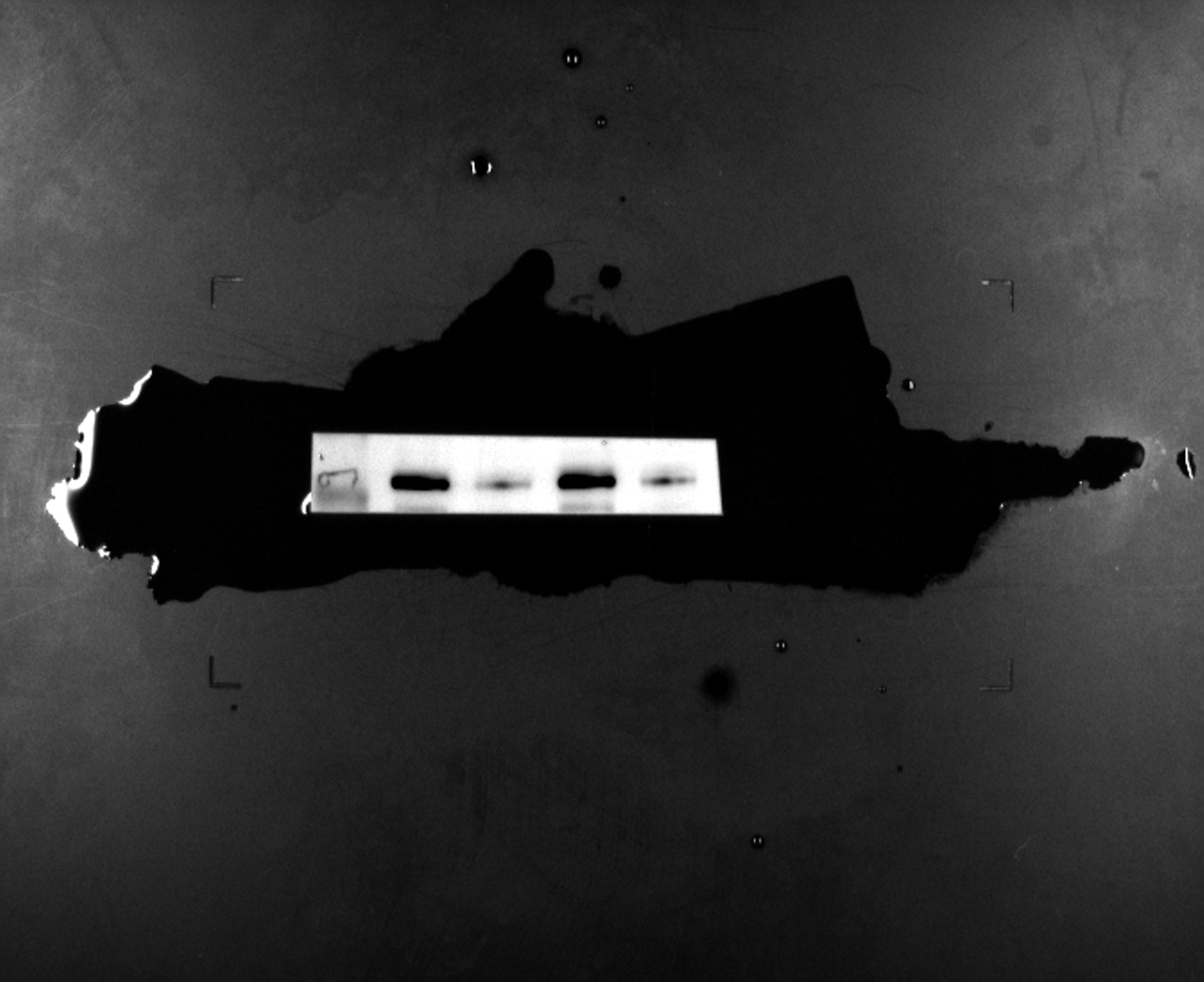


Actin


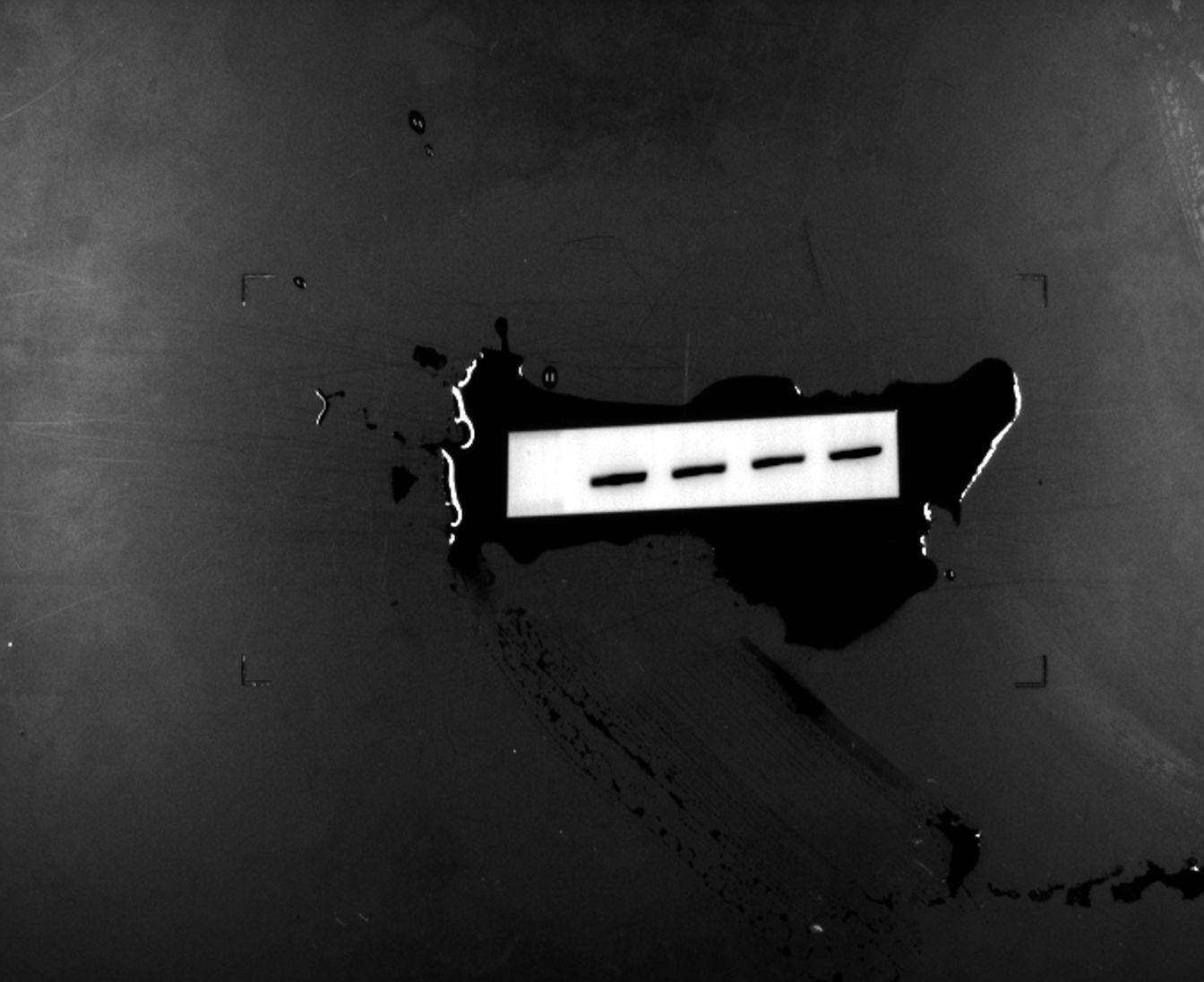


Fig 2D

NR4A1


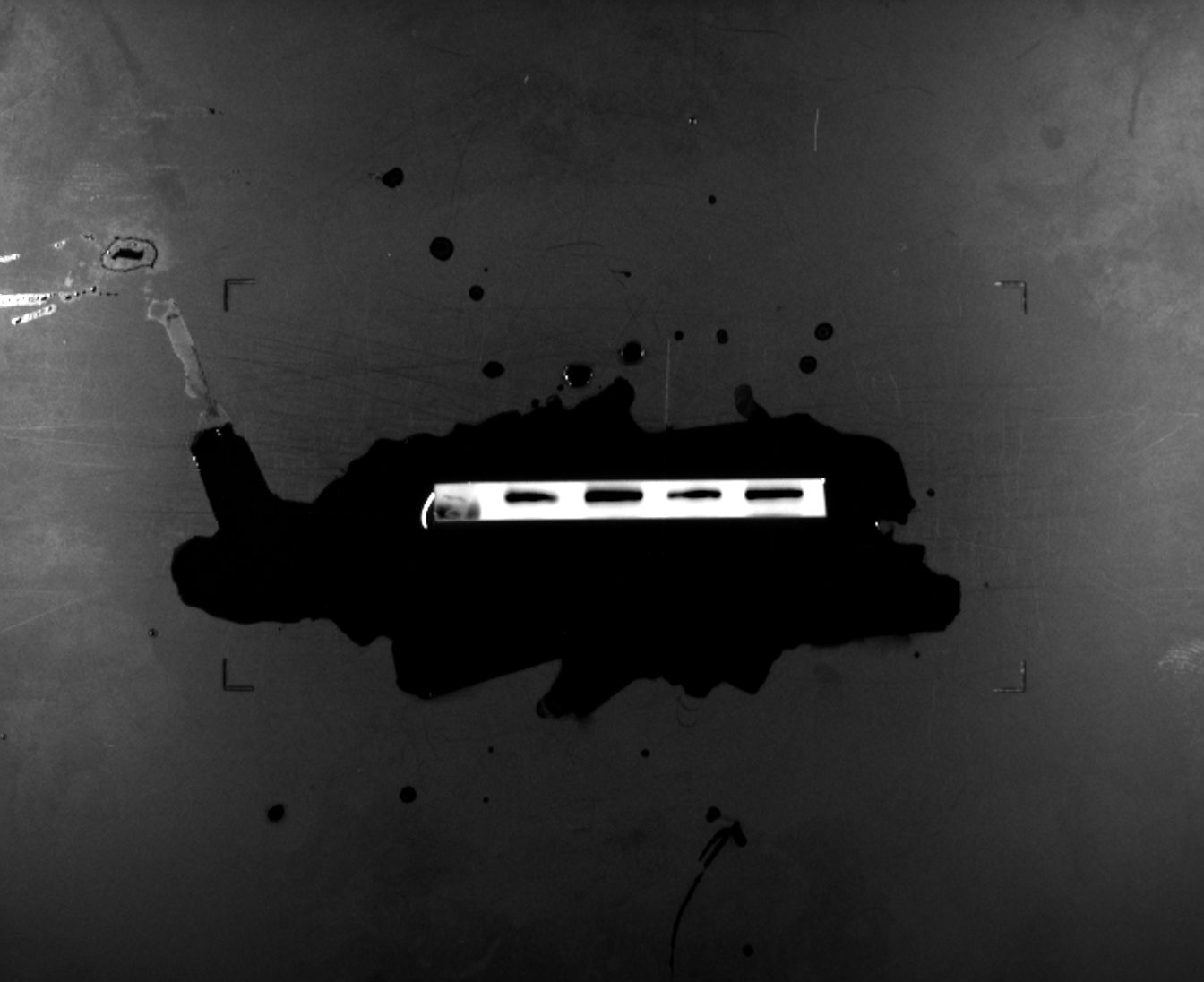


Actin


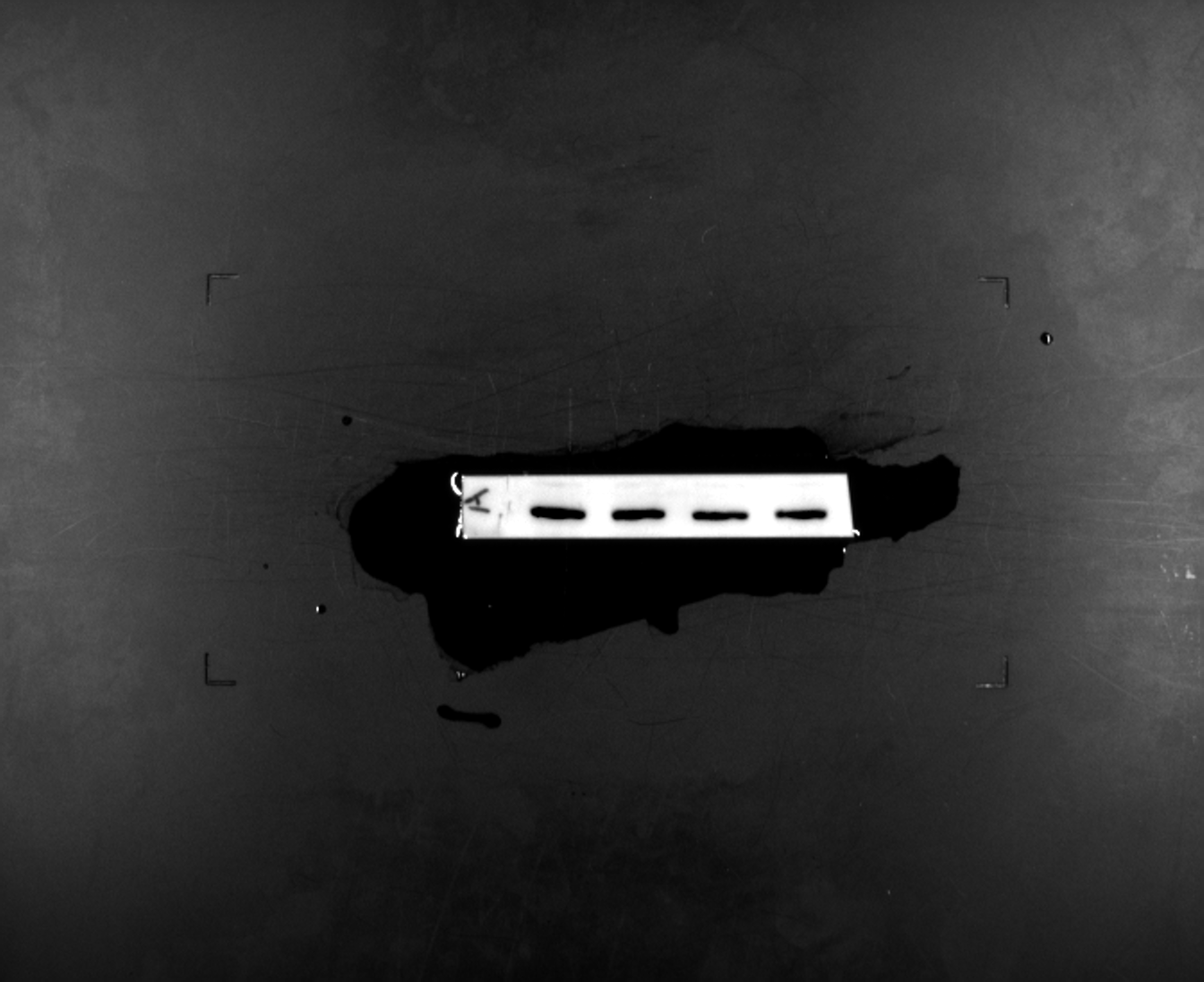


Fig2J

γ-H2AX


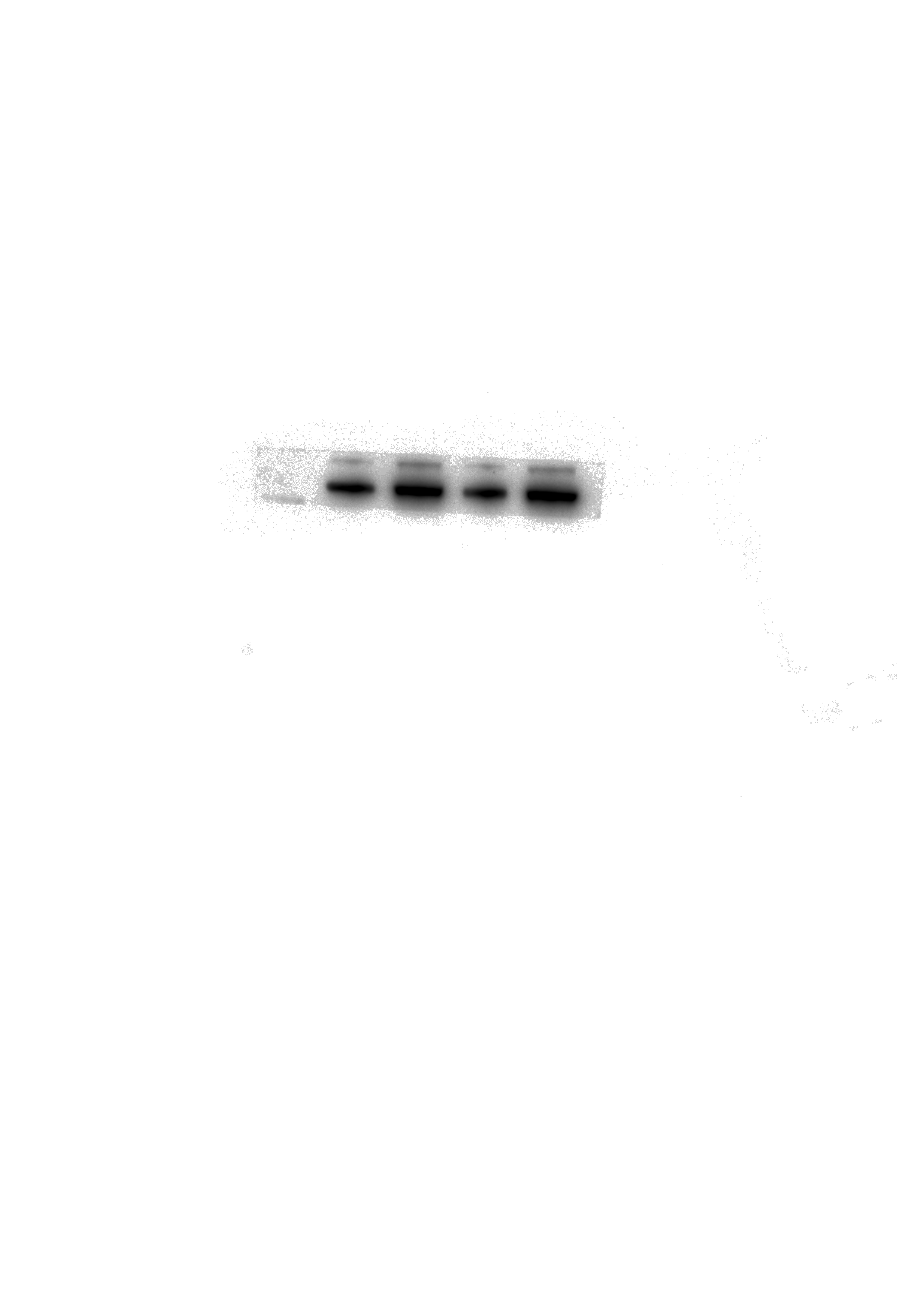


P53


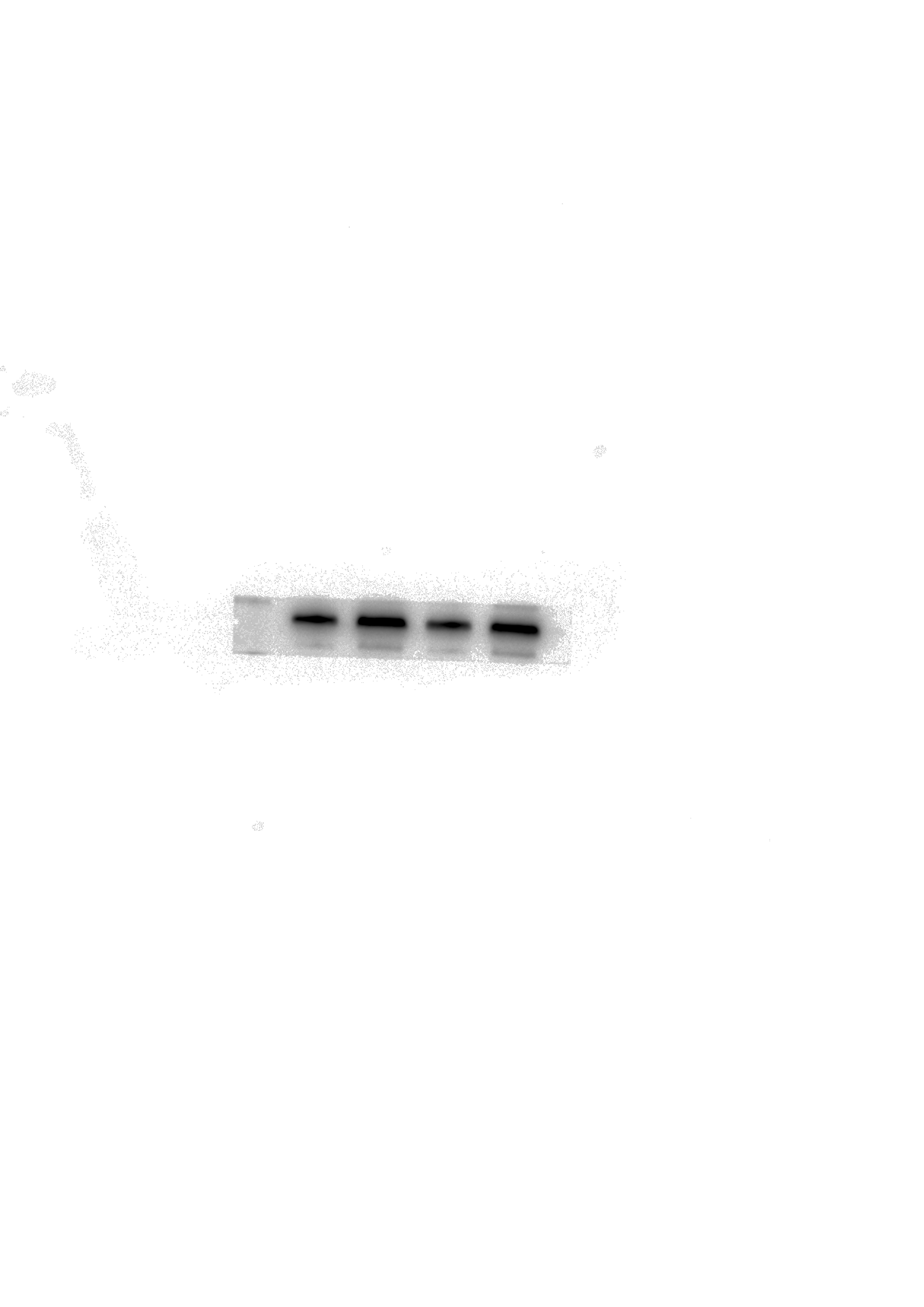


Bax


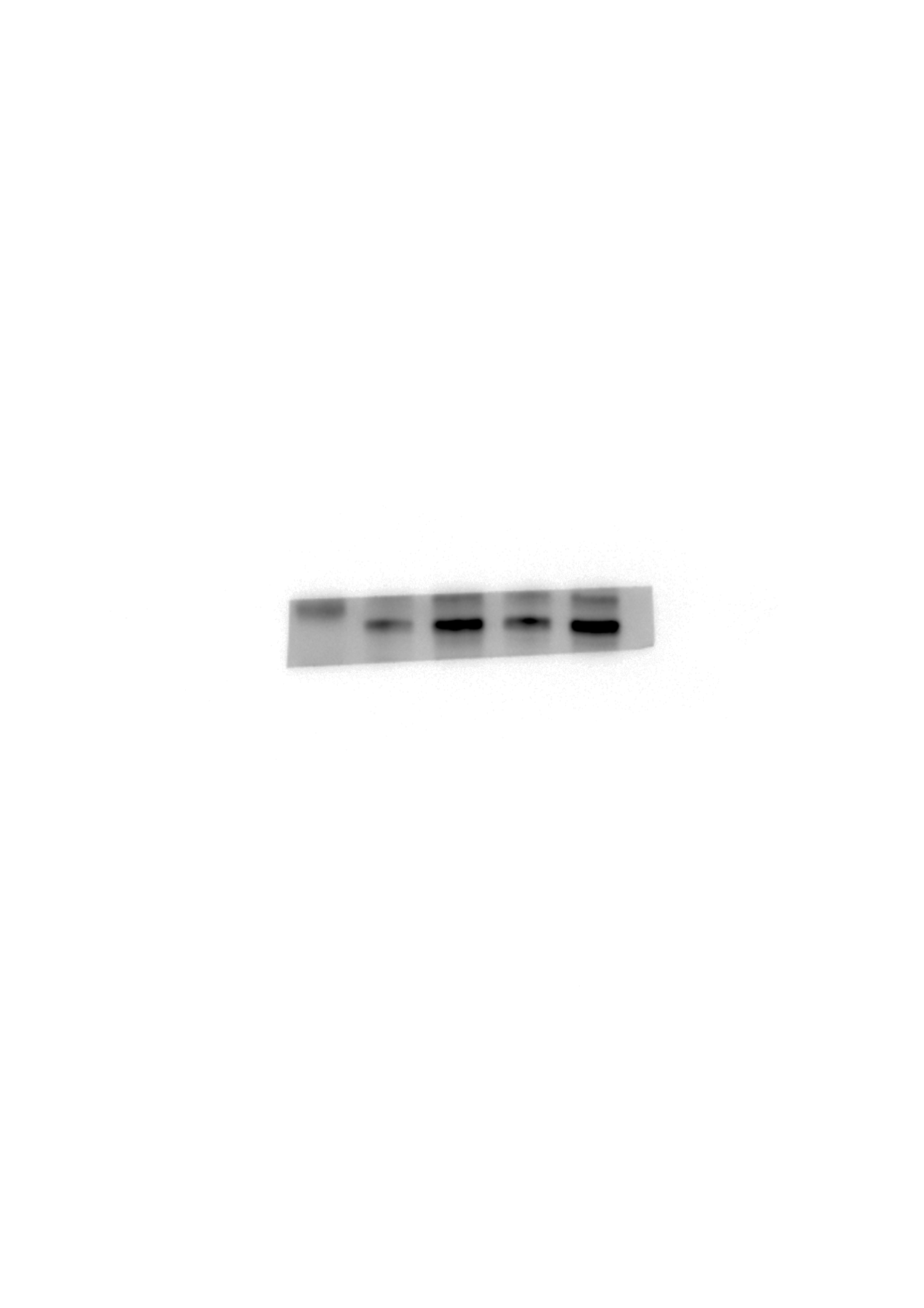


Caspase3


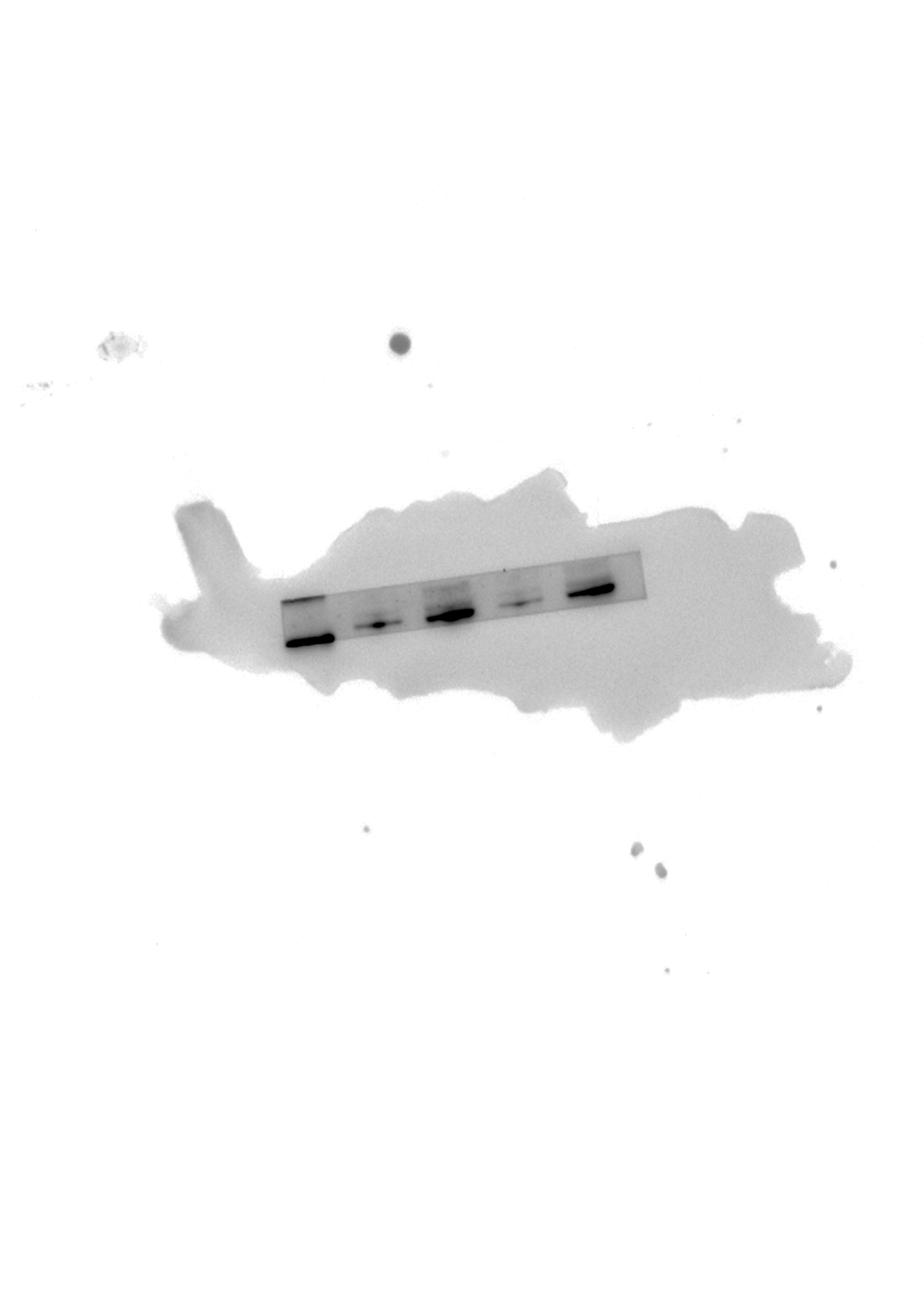


Bcl-2


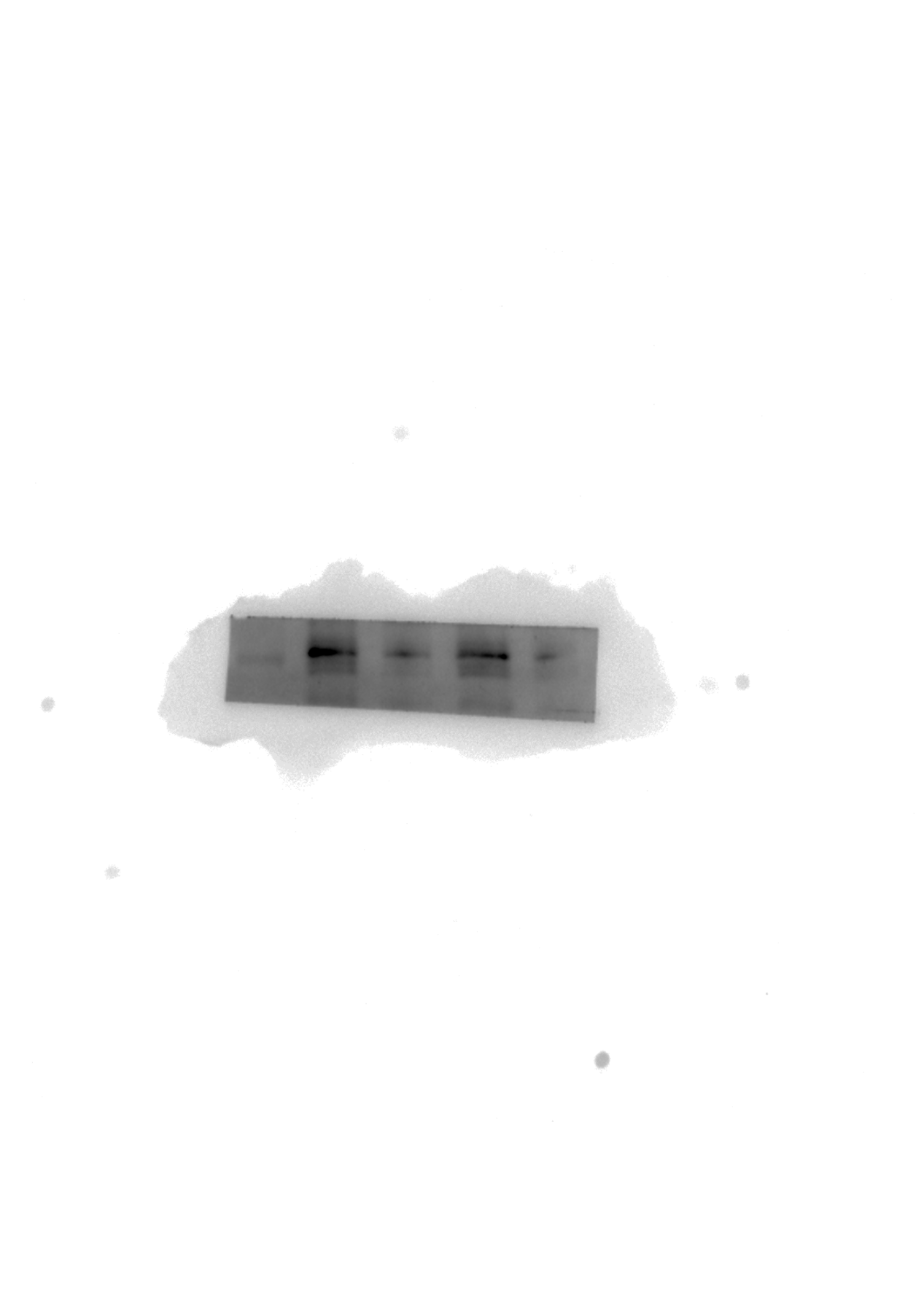


Actin


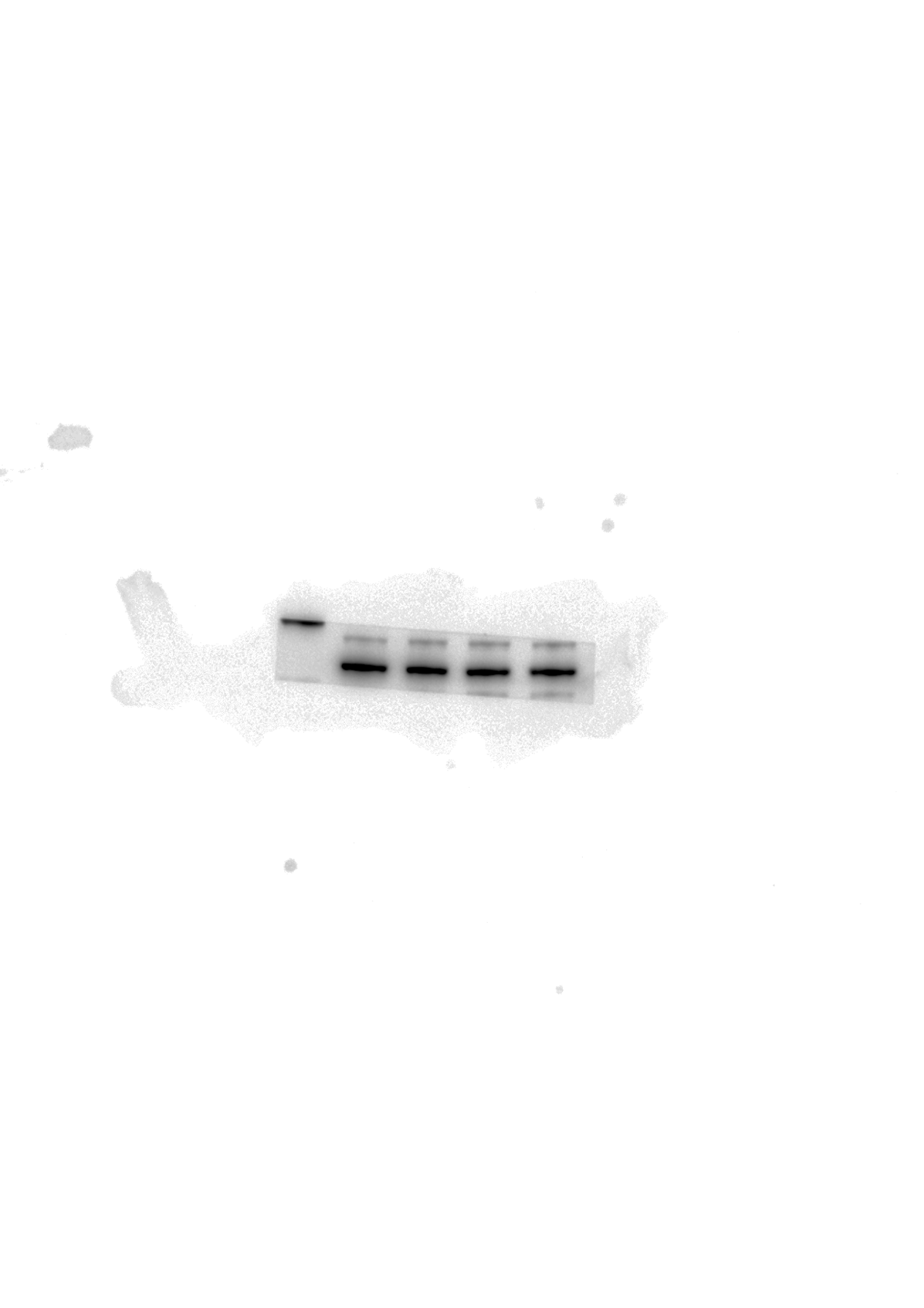


Fig3 G

E-cadherin


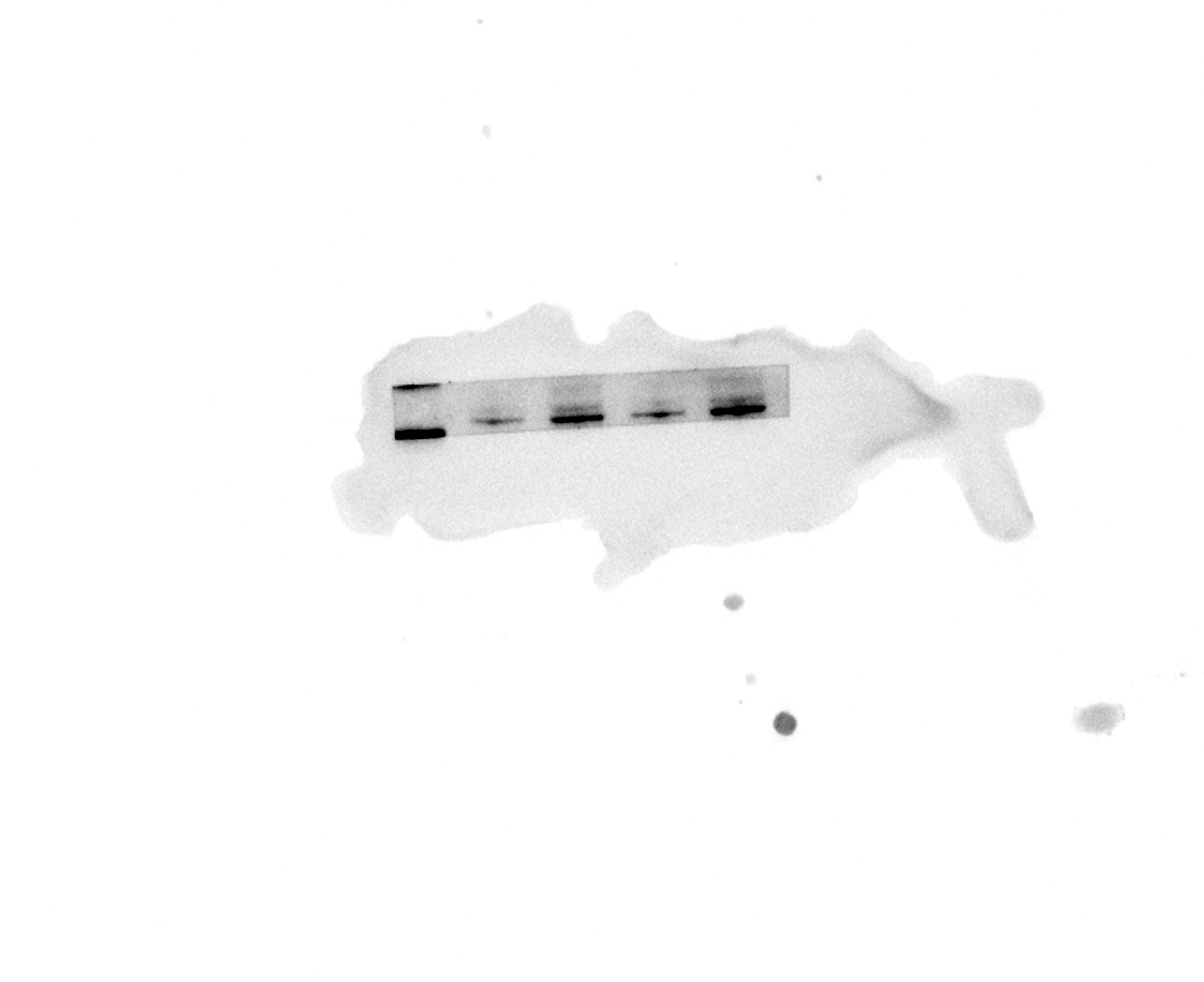


1. cadherin

Vimentin
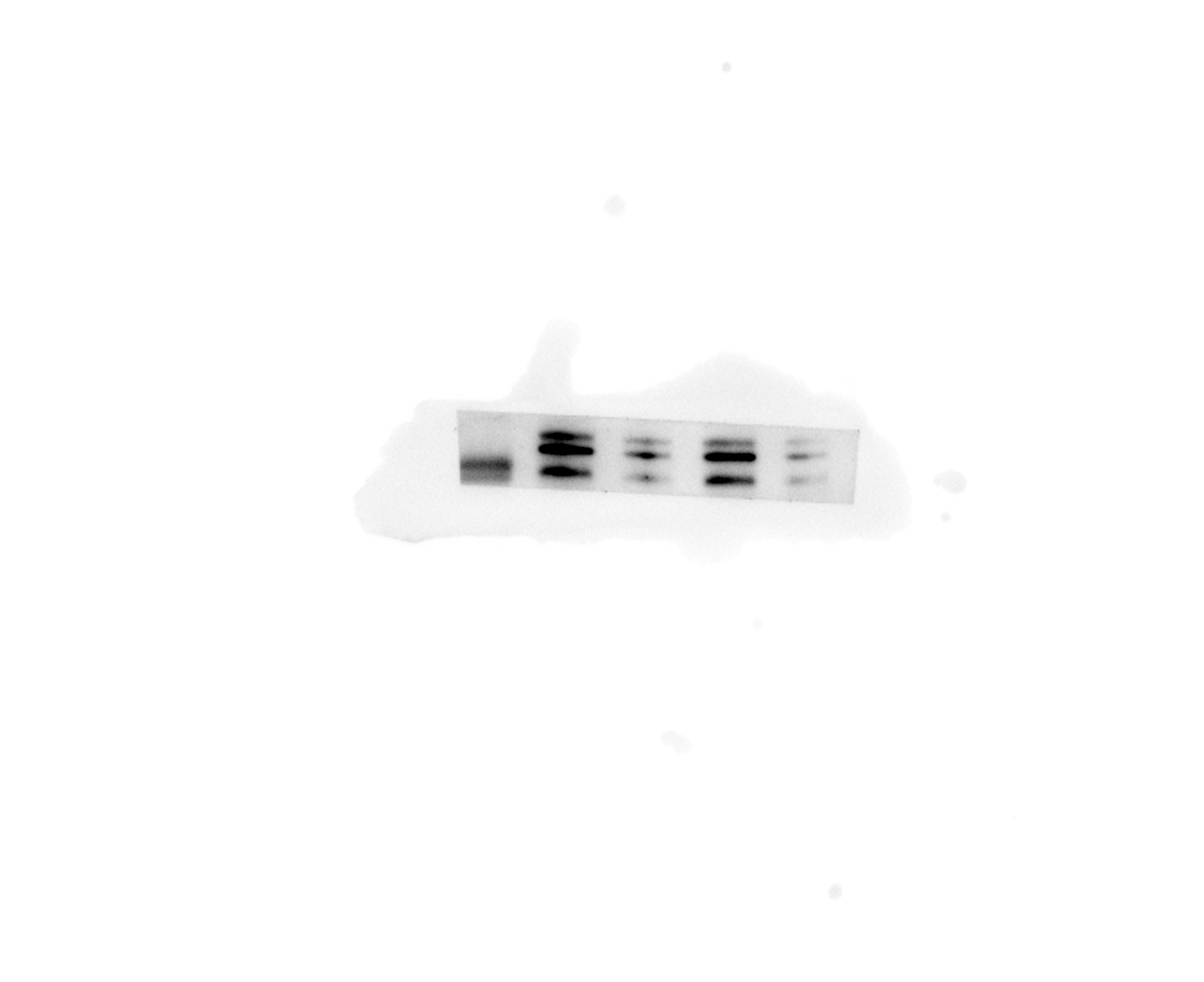

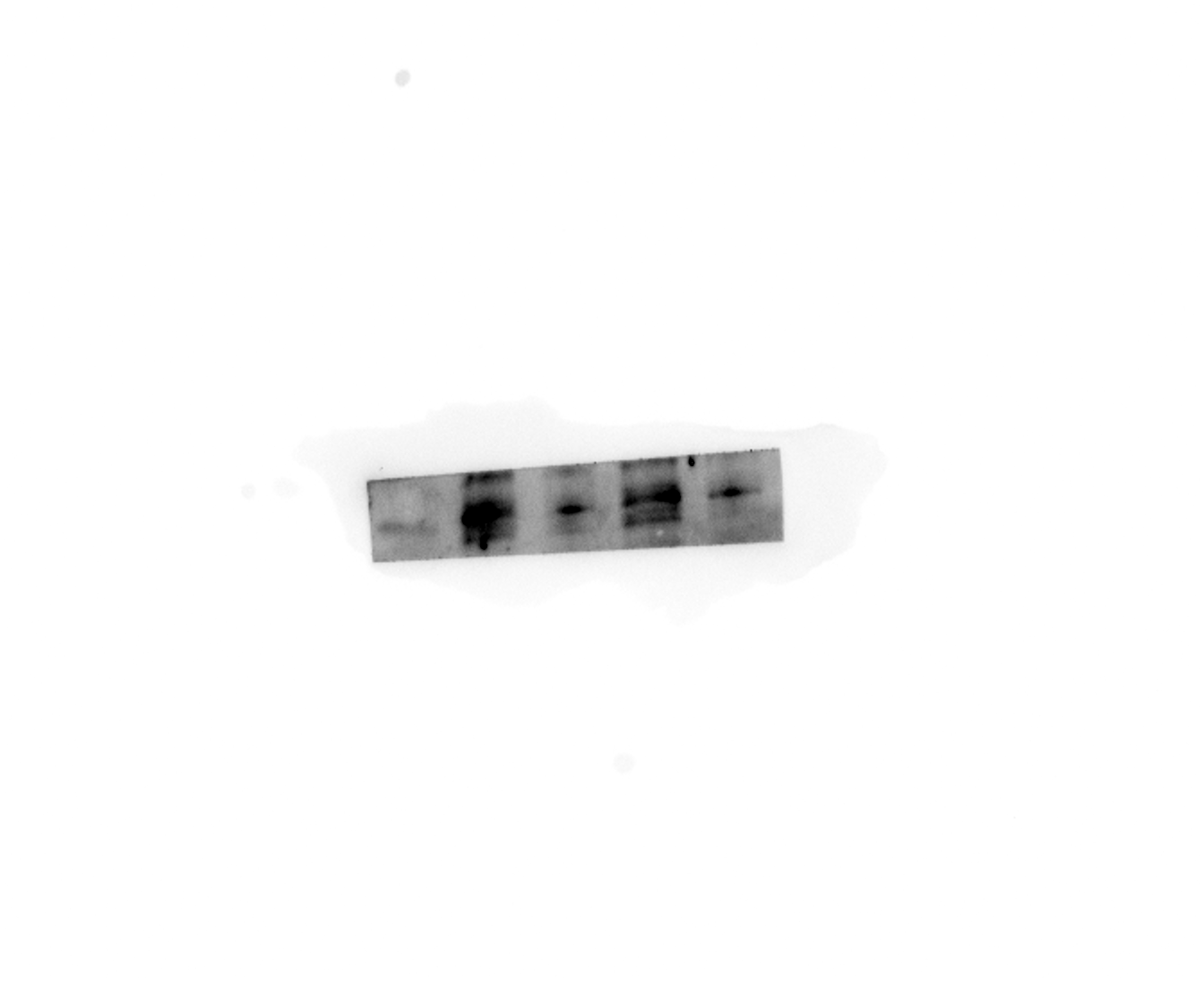


Actin


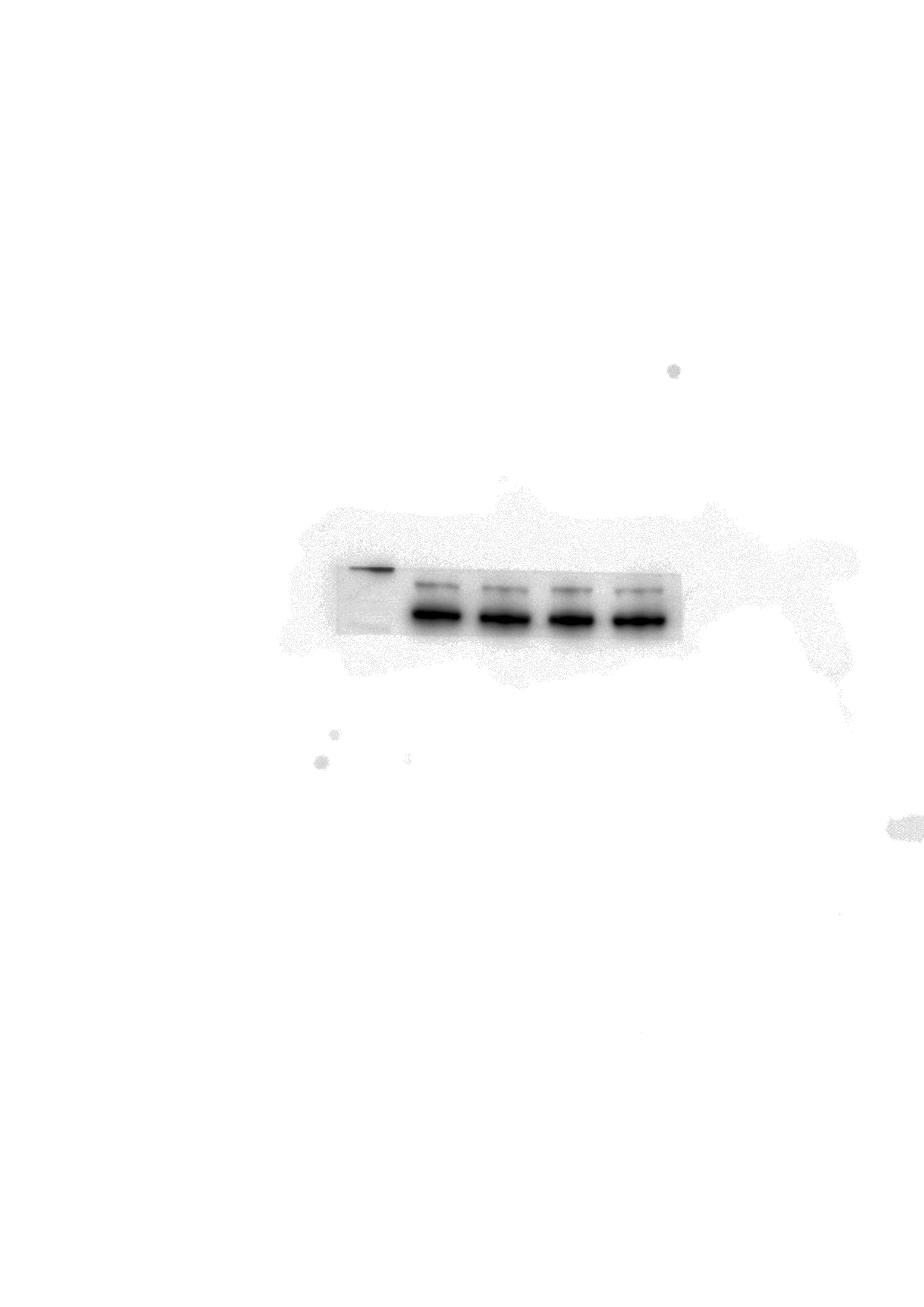

Supplement: Supplementary file 3 — Supplementary Material 3 [file 41598_2025_20348_MOESM3_ESM.docx]
